# Recent Advances in Endometrial Cancer Prevention, Early Diagnosis and Treatment

**DOI:** 10.3390/cancers16051028

**Published:** 2024-03-01

**Authors:** Holly Baker-Rand, Sarah J. Kitson

**Affiliations:** 1 Division of Cancer Sciences, Faculty of Biology, Medicine and Health, University of Manchester, Manchester M13 9PL, UK; holly.baker-rand@manchester.ac.uk; 2 Manchester Academic Health Science Centre, Department of Obstetrics and Gynaecology, Manchester University NHS Foundation Trust, Manchester M13 9WL, UK

**Keywords:** endometrial cancer, prevention, diagnosis, treatment, advances

## Abstract

**Simple Summary:**

Endometrial cancer is the fourth most common female malignancy in high socioeconomic index nations and the sixth most common cancer in women worldwide. The disease incidence has increased globally by 132% in the last 30 years, and this trend is set to continue in light of an ageing population and increasing levels of obesity and diabetes. Although more women than ever before are dying of endometrial cancer, the mortality rates are falling due to recent advances in early diagnosis and treatment. Our understanding of the molecular drivers of endometrial cancer has increased substantially, and doctors are now, for the first time, starting to translate this knowledge into the true personalisation of care. This review summarises the recent advances in the field and identifies avenues for future exploration.

**Abstract:**

Endometrial cancer is the sixth commonest cancer in women worldwide, with over 417,000 diagnoses in 2020. The disease incidence has increased by 132% over the last 30 years and is set to continue to rise in response to an ageing population and increasing global rates of obesity and diabetes. A greater understanding of the mechanisms driving endometrial carcinogenesis has led to the identification of potential strategies for primary disease prevention, although prospective evaluation of their efficacy within clinical trials is still awaited. The early diagnosis of endometrial cancer is associated with improved survival, but has historically relied on invasive endometrial sampling. New, minimally invasive tests using protein and DNA biomarkers and cytology have the potential to transform diagnostic pathways and to allow for the surveillance of high-risk populations. The molecular classification of endometrial cancers has been shown to not only have a prognostic impact, but also to have therapeutic value and is increasingly used to guide adjuvant treatment decisions. Advanced and recurrent disease management has also been revolutionised by increasing the use of debulking surgery and targeted treatments, particularly immunotherapy. This review summarises the recent advances in the prevention, diagnosis and treatment of endometrial cancer and seeks to identify areas for future research.

## 1. Introduction

The endometrial cancer landscape has changed markedly over the last 30 years, with a notable rise in disease incidence and increasing patient complexity. In 2020, there were over 417,000 new cases of endometrial cancer diagnosed worldwide and 97,370 disease-related deaths, representing a substantial health burden [1]. These numbers are set to increase further in light of an ageing global population and rising rates of obesity and diabetes [2]. A better understanding of the mechanisms through which these risk factors drive endometrial carcinogenesis has provided an opportunity to develop targeted interventions for primary disease prevention, with the aim of halting this increase. Although the number of endometrial cancer deaths has nearly doubled since 1990, the age-standardised mortality rate has significantly decreased in almost all global regions, reflecting advances in early diagnosis and treatment [3]. Whilst the majority of women are diagnosed with early-stage disease and can expect to be cured of their endometrial cancer, up to 20% of women continue to have extra-uterine disease at presentation, with only 15% of women with stage IV disease alive five years after diagnosis [2]. Early diagnosis, therefore, remains imperative, and non-invasive tests designed to exclude endometrial cancer in the majority of women with postmenopausal bleeding are rapidly increasing in number and are likely to be more acceptable to women than endometrial biopsies. Of concern is the widening disparity in survival between White, Black, Asian and Hispanic women, which may be related as much to the lack of access to endometrial cancer treatments as the differences in tumour biology [4,5]. A greater understanding of the genetic changes driving endometrial carcinogenesis will hopefully be used to address this gap, but this has already begun to be used to personalise treatments and to identify those women with Lynch syndrome, for whom their endometrial cancer represents a sentinel malignancy [6]. This review summarises these recent advances in endometrial cancer prevention, diagnosis and treatment and seeks to identify the outstanding research questions to be addressed in the next 30 years.

## 2. Advances in Endometrial Cancer Prevention

Given the strong association with modifiable risk factors, endometrial cancer appears eminently suited to primary disease prevention, with modelling suggesting that up to 60% of endometrial cancer cases could be potentially prevented [7,8,9]. An increased understanding of the mechanisms driving endometrial carcinogenesis, namely unopposed oestrogen, insulin resistance and chronic inflammation, has led to the proposal of a number of interventions designed to reduce endometrial cancer incidence, albeit with data on their efficacy largely limited to retrospective observational studies [10,11].

### 2.1. Weight Management

Obesity has the strongest link with endometrial cancer of the twenty most common tumour types and is plausibly implicated in 34% of diagnoses [7]. Conversely, weight loss, by reducing adiposity and the aromatase-induced conversion of androgens into oestrogen, improving insulin sensitivity and lowering the levels of inflammation, is associated with a reduction in the endometrial cancer risk [11]. Observational data from the Women’s Health Initiative suggests that intentional weight loss of as little as 5% bodyweight over a three year period is associated with a 39% (95%CI 12–58%) reduction in endometrial cancer incidence [12]. Interestingly, whilst women with obesity had the greatest benefit (HR 0.44, 95%CI 0.25–0.78), weight loss was also beneficial for women with a BMI within the normal range (HR 0.61, 95%CI 0.27–1.38). Achieving weight loss through lifestyle modification is feasible, but challenging to maintain and risks weight cycling, which appears to be more detrimental to endometrial cancer risk than a stable higher weight [13]. Certainly, the seven hours of jogging a week required to reduce endometrial cancer risk by 10% is not likely to be achievable for the majority of women [14].

The focus has, therefore, shifted to the new anti-obesity medications (AOMs) that have been developed in recent years and which appear to lead to more significant and sustained weight loss in comparison with lifestyle interventions and older AOMs such as orlistat [15,16]. Glucagon-like peptide 1 (GLP-1) agonists, such as semaglutide and liraglutide, improve insulin sensitivity, delay gastric emptying and decrease a person’s appetite [17]. The mean weight change at two years was −15.2% with weekly semaglutide compared with −2.6% with a placebo in a STEP 5 randomised controlled trial (RCT), when used in combination with a behavioural intervention (*p* < 0.0001) [18]. Liraglutide appears to be similarly efficacious, with 51.8% of participants achieving ≥5% bodyweight loss compared with 24% of those treated with a placebo in an SCALE RCT (*p* < 0.0001) [19]. The side effects include gastrointestinal disruption, skin reactions, atrioventricular blockage, and rarely, pancreatitis, and long-term tolerance and safety have yet to be established. Whether the effects of GLP-1 agonists on bodyweight and insulin sensitivity can also translate into a reduction in endometrial cancer risk remains to be seen and should be included as a secondary outcome measure in long-term cohort studies.

Bariatric surgery alters the anatomy of the digestive system to restrict the capacity of the stomach, reduce nutrient absorption and induce early satiety [20]. It is the most effective intervention for obesity identified to date, leading to the long-term weight loss of up to 29 kg depending upon the exact procedure performed [21]. Wilson et al. demonstrated in their meta-analysis that bariatric surgery is effective in reducing the risk of subsequent endometrial cancer by 62% (pooled relative risk 0.38, 95%CI 0.26–0.55) [22]. Despite this, endometrial cancer prevention is not currently an indication for bariatric surgery, potentially because of the risk of long-term complications, including malabsorption, nutritional deficiencies, small bowel obstruction, dumping syndrome and gastric or stomal stenosis and resource issues limiting its availability [23].

### 2.2. Hormonal Chemoprevention

The beneficial effects of oral contraceptives on endometrial cancer risk have been known for the last 20 years, with every five years of use associated with a 24% reduction in the disease risk [24]. Importantly, this effect appears to persist for up to 30 years after the discontinuation of use. Whilst oestrogen-containing preparations are not advisable for women with a BMI > 35 kg/m^2^ due to an elevated risk of arterial and venous thrombotic events, the combined oral contraceptive pill is recommended by the international consensus group for women with Lynch syndrome requiring contraception due to its beneficial effects on both the endometrial and ovarian cancer risks [25,26]. Progestin-only contraception is also likely to be beneficial, although the discontinuation rates are often higher due to irregular bleeding [27]. The levonorgestrel-releasing intra-uterine system (LNG-IUS) may well be the most effective endometrial cancer prevention measure, with large-scale observational studies describing up to a 78% reduction in endometrial cancer risk among the users, particularly if used long-term [28,29]. It certainly appears to reduce endometrial proliferation, even in women with a BMI ≥ 40 kg/m^2^, with modelling suggesting that it could be a cost-effective approach for primary disease prevention in high-risk women [30,31]. Reassuringly, the previously raised concerns about an increased risk of postmenopausal breast cancer with LNG-IUS use were not confirmed in a recent meta-analysis [32]. Adequately powered clinical trials are now required to determine whether the LNG-IUS is both effective at reducing the incidence of endometrial cancer and is sufficiently acceptable to women for it to be used in routine practice.

### 2.3. Aspirin

Aspirin, a cycloxyengase-2 inhibitor, has anti-inflammatory effects and acts to reduce the aromatase and oestrogen levels and increase apoptosis [33,34]. Whilst women within the general population may benefit from only a small reduction in endometrial cancer risk from long-term aspirin use (8–11%), this may prove to be a more effective strategy in women with obesity (relative risk reduction 20–44%) [35]. The CAPP2 study demonstrated a clear reduction in the colorectal cancer risk with regular aspirin use in individuals with Lynch syndrome, but this RCT was insufficiently powered to assess the benefit to women with regard to the endometrial cancer risk [36]. The results were, however, encouraging (hazard ratio 0.50, 95%CI 0.22–1.11) and should be investigated further.

### 2.4. Metformin

Much interest has been expressed in the re-purposing of metformin, an oral biguanide and insulin-sensitiser, in the management of endometrial cancer. Despite early promising results in single-arm studies, metformin did not appear to reduce endometrial proliferation when evaluated within a more methodologically robust RCT [37]. These findings have been confirmed in a Cochrane review, in which it was noted that there was insufficient evidence to support the use of metformin either alone or in combination with progestin therapy for the management of endometrial hyperplasia [38]. The authors did note, however, that only two trials totally 59 patients were eligible for inclusion in their review, making it difficult to draw generalisable conclusions. A meta-analysis of six studies also found that metformin use was not associated with a reduction in the endometrial cancer risk (odds ratio 1.05, 95%CI 0.82–1.35), even when adjusting for the confounding variable of diabetes [39]. A recently published feMMe trial again demonstrated the limited cytostatic effect of metformin on the endometrium, with no increase in the effectiveness of the LNG-IUS for the management of early-stage endometrial cancer with the addition of metformin [40]. Together, these results should dissuade researchers from pursuing metformin for the chemoprevention or treatment of endometrial cancer.

### 2.5. Identifying High-Risk Women

Identifying high-risk individuals for targeted endometrial cancer prevention is imperative in order to maximise the benefits and minimise the risk of harm from long-term intervention. Women with Lynch syndrome represent the highest-risk group, with a 40–60% lifetime risk of endometrial cancer depending upon the underlying genetic variant [41]. The widespread adoption of reflex immunohistochemistry for mismatch repair genes (*MLH1*, *MSH2*, *MSH6* and *PMS2*) on endometrial cancers prompts the identification of affected individuals, who will, themselves, benefit from surveillance for colorectal malignancies and whose affected female relatives should be offered a risk-reducing hysterectomy from the age of 40 years [26].

Only 3% of endometrial cancers are, however, related to Lynch syndrome, with a much greater number of women within the general population at moderate–high risk of the disease secondary to polygenic and environmental factors. Offering an intervention to this group has the potential to dramatically reduce the endometrial cancer incidence. To date, four risk prediction models have been developed with the aim of stratifying the endometrial cancer risk within the general population, of which three models have been externally validated [42,43,44,45]. All are based on similar epidemiological risk factors, with or without the addition of serum biomarkers, with the E2C2 and PRECISION models demonstrating the highest discrimination (0.64–0.69) and best calibration (E/0 1.03–1.09) via external validation [44,45]. The advantage of the PRECISION model is its generalisability to non-White ethnic groups and suitability for use in both pre- and postmenopausal women, although, currently, it has only been validated within a UK population. In this setting, it outperformed the other published models in decision curve analysis and was associated with a greater net benefit than offering endometrial cancer prevention to all the women. An endometrial cancer risk assessment appeals to women who are keen to know their own individualised risk, in theory, and are willing to make lifestyle changes or have an LNG-IUS inserted if it will be of benefit [46]. The uptake of interventions to reduce the endometrial cancer risk need, therefore, to be urgently assessed within the context of a clinical trial.

## 3. Advances in Early Diagnosis

The majority of cases of endometrial cancer are diagnosed among postmenopausal women who frequently present with postmenopausal bleeding [10]. Transvaginal ultrasound in combination with endometrial sampling enables the histological diagnosis of endometrial cancer and can accurately exclude the presence of disease. Endometrial biopsies are, however, invasive and have a failure rate of around 11% due to inadequate samples and cervical stenosis [47]. Innovations in diagnostic tests are underway to identify alternative methods of identifying high-risk women for further testing and which can be used to reassure those at low risk of the disease. Peripheral blood, cervicovaginal fluid and urine offer potential sources of DNA or protein biomarkers and cells for cytological assessment.

### 3.1. Peripheral Blood

Human epididymis protein 4 (HE4) has emerged as the most promising of a number of serum diagnostic endometrial cancer biomarkers examined to date. Two meta-analyses have demonstrated a relatively modest pooled sensitivity of 65%, but with a higher specificity at 91%, albeit with marked inter-study heterogeneity [48,49]. The addition of further biomarkers, including cancer antigen 125 (CA125), and anthropometric data, such as BMI, has not been shown to improve the test performance [50]. These results suggest that the HE4 levels alone are unlikely to be sufficiently informative for them to be used in a diagnostic test, but that they may have a role as a triage tool for high-risk populations.

There has been increasing interest in the role of plasma cell free DNA (cfDNA), circulating tumour DNA (ctDNA) and circulating microRNA (miRNA) for the detection of endometrial cancer. The next-generation sequencing of cfDNA using a targeted four-gene panel (CTNNB1, K-ras, PTEN and PIK3CA) identified mutations within the plasma that matched those within the corresponding endometrial tumour in 33% of women, although this could only detect 18% of mutations in women with early-stage disease [51]. A recent study using ctDNA detected the hypermethylation of *zinc finger and SCAN domain containing 12* (*ZSCAN12*) and/or *oxytocin* (*OXT*) in 9/11 and 5/20 women with advanced and non-advanced endometrial cancer, respectively, giving a sensitivity of 98%, specificity of 97% and an area under the curve (AUC) of 0.99 [52]. Fan et al. assessed the miRNA signatures in 92 endometrial cancer subjects and 102 control subjects and externally validated the identified signatures in three large datasets. Six miRNAs (miR-143-3p, miR-195-5p, miR-20b-5p, miR-204-5p, miR-423-3p and miR-484) were overexpressed in endometrial cancer, with an AUC via external validation of 0.97 [53]. The observed discrepancy in test performance based on the stage of disease is not surprising given that localised disease is unlikely to shed sufficient ctDNA into the blood for it to be reliably detected. These biomarkers may, therefore, be better suited to identifying aggressive and potentially recurrent endometrial cancer rather than being used for the early diagnosis of disease.

Advances in the use of high-throughput technology have led to a rapid increase in the number of registered studies aiming to identify novel genomic, transcriptomic, proteomic and metabolomic blood biomarkers to be used in the early diagnosis of endometrial cancer. Single biomarkers may not be sufficiently informative to be used in clinical practice, and many of the ‘omic signatures’ identified have yet to be externally validated. This remains, however, an active area of research.

### 3.2. Uterine and Cervicovaginal Fluid

Uterine lavage fluid has been shown to have good sensitivity and specificity for endometrial cancer detection; however, it needs to be collected via hysteroscopy and is associated with significant discomfort for women [54]. Cervicovaginal samples are easier to obtain and can be as informative for the detection of an endometrial cancer as uterine lavage due to the shedding of malignant cells into the lower genital tract. The PapSEEK test, which incorporates assays for mutations in 18 genes as well as aneuploidy, has been shown to identify 81% of endometrial cancers when conducted during a routine Pap test, including 78% with early-stage disease [55]. The sensitivity increased to 93%, with a specificity of 100% when a Tao brush was used to obtain the samples. The Tao brush needs to be inserted up to the uterine fundus though and is associated with discomfort and a high failure rate. Alternatively, vaginal tampons can be used to collect cervicovaginal fluid, representing an inexpensive, easy-to-use self-collection system that is more likely to appeal to women. The samples can be analysed by next-generation sequencing to identify somatic mutations [56] or, of notable promise, undergo methylation testing [57]. The WID-qEC test, a three-marker test that appraises DNA methylation in the gene regions of GYPC and ZSCAN12 has been shown to outperform the transvaginal ultrasound measurement of endometrial thickness in the diagnosis of endometrial cancer (AUC WID-qEC 0.94 vs. ET measurement 0.87) among women with abnormal uterine bleeding [57]. The high negative predictive value of the test means that it could be used to reduce the number of women requiring invasive histological assessments.

### 3.3. Urine and Vaginal Cytology

Intact cells shed from the upper genital tract can also be detected in the vaginal fluid and, interestingly, within urine. This is thought to be related to the contamination of voided urine samples during self-collection, with higher cellular loads potentially more likely to be found in the samples collected from women with concurrent abnormal uterine or postmenopausal bleeding [58]. Cytological assessment is labour-intense and requires specialist interpretation, but has been shown within a diagnostic accuracy study to have a combined sensitivity of 91.7% (95%CI 85.0–96.1%) and specificity of 88.8% (95%CI 81.2–94.1%) for the detection of gynaecological malignancies, the majority of which were endometrial cancers [58]. The high negative predictive value (91.4%, 95%CI 84.9–95.2%) of urine and vaginal cytology may mean that it can be used to identify women for future investigations, reducing the number of women with abnormal uterine bleeding who require an invasive endometrial biopsy. There is clear potential for the analysis of these samples to extend to adjunct immunocytochemistry and genomic analysis, with the role of artificial intelligence to support this currently under investigation.

All of these novel diagnostic techniques have the potential to change the landscape of endometrial cancer diagnosis. Minimally invasive tests are likely to reduce pain and distress for women and could be used to reassure women at low risk of the disease, meaning that only those most likely to have an underlying endometrial cancer are required to undergo endometrial biopsy. These tests could also be used repeatedly to screen for endometrial cancer in women at high risk of the disease. At present, however, these early diagnostic techniques are only being used within the context of research studies, with evidence of their clinical utility available from a limited number of small cohorts of women who have already been diagnosed with endometrial cancer or presenting with postmenopausal bleeding. Large prospective multicentre trials are urgently needed to confirm the diagnostic accuracy of these techniques if they are to be rolled out into routine clinical practice.

## 4. Surgery

Sentinel lymph node mapping has transformed surgery for endometrial cancer since it was first described by Burke et al. in 1996 [59]. Whilst the first pilot study involved the use of isosulfan blue dye injected into the subserosal myometrium, the more recent studies have used a variety of tracers alone or in combination (blue dye, indocyanine green (ICG) and technetium-99m) and a number of different routes of injection (subserosal, cervical and endometrial via hysteroscopy). A Cochrane review failed to demonstrate the significant effect of type or route of dye injection on the number of sentinel lymph nodes identified, although the authors noted that the number of studies using the same protocol that could be combined in a meta-regression analysis was small [60]. Accessing the cervix for injection is certainly easier than pursuing peri-tumoural injection, and ICG may offer an improved sentinel lymph node detection rate over that of blue dye [61]. This certainly has the added advantage of compatibility with robotic platforms now in widespread use. Given the high detection rate (mean 86.9%, 95%CI 82.9–90.8%) and sensitivity (pooled 91.8%, 95%CI 86.5–95.1%) of sentinel lymph node detection secondary to ultrastaging combined with a low false-positive rate and reduced risk of lymphoedema, many international societies have recommended sentinel lymph node biopsy replace pelvic node dissection as part of endometrial cancer staging [60,62,63]. Whilst a therapeutic benefit from pelvic lymphadenectomy in women with presumed early-stage endometrial cancer has never been demonstrated, it does allow for adjuvant treatment to be used selectively among those at the greatest risk of disease recurrence [64].

Robotic surgery is now much more widely available and is being used increasingly to treat women with endometrial cancer. Despite the concerns about cost and longer operating times, it does appear to offer a number of advantages over conventional (straight-stick) laparoscopy, particularly in women with a BMI ≥ 40 kg/m^2^. In particular, its use is associated with a shorter hospital stay and lower rate of conversion to laparotomy (robotic surgery pooled conversion rate 3.8%, 95%CI 1.4–9.9 vs. laparoscopic surgery 7.0%, 95%CI 3.2–14.5), potentially because of the use of lower intra-abdominal pressure and consequent reduced intolerance of the Trendelenburg position [65,66]. Importantly, similar oncological outcomes have been demonstrated for patients undergoing robotic, laparoscopic and open surgery for early-stage endometrial cancer in retrospective series, although the data from prospective longitudinal studies are still awaited [66,67]. There are also benefits for surgeons, with improved ergonomics and reduced muscle usage, especially when operating on patients with a high BMI [68].

Whilst surgery remains the primary treatment modality for women diagnosed with early-stage endometrial cancer, the role of cytoreductive surgery in advanced disease continues to be contentious. Barlin et al. demonstrated in their meta-analysis of 14 retrospective cohort studies that a 10% increase in the complete cytoreduction rate for women with advanced endometrial cancer was associated with a 9.3-month increase in overall survival [69]. Whether the presence of any residual disease at the end of surgery negatively impacts upon survival remains under debate, largely because of the widely varying definitions used to describe optimal cytoreduction in the literature [69,70]. Over the last 15 years there has been an increase in the use of neoadjuvant chemotherapy in the treatment of advanced endometrial cancer, with the hope of trying to improve on the otherwise disappointing rates of maximal cytoreduction with primary surgery [70]. There are, however, only limited data demonstrating a reduction in surgical morbidity and operating time and a modest improvement in rates of optimal cytoreduction with this approach [71]. This is perhaps not surprising given the diverse patients and surgical complexity being studied. Evidence on the long-term oncological outcomes of women undergoing neoadjuvant chemotherapy and interval surgery for advanced endometrial cancer should be a research priority.

## 5. Adjuvant Treatment

Despite the lack of prospective clinical trial data on the use of molecular profiling to determine management, the FIGO have elected to incorporate the four endometrial cancer molecular subgroups (POLE*mut*, MMRd, NSMP and p53abn) into their most recent staging [72,73]. The implementation of the new system has the potential to lead to profound changes in the adjuvant treatment of endometrial cancer, but we should await the results of an ongoing multi-arm RAINBO trial [74]. This umbrella study consists of three randomised controlled trials and one prospective two arm phase II trial and will compare radiotherapy and chemotherapy with and without the maintenance of Olaparib (p53*abn*), radiotherapy alone or in combination with durvalumab (MMRd), radio- and chemotherapy with radiotherapy and progesterone maintenance (NSMP) and radiotherapy with no additional treatment (POLE*mut*) in an adjuvant setting. Survival and quality of life outcomes at three and five years will be assessed.

In the interim, the molecular characterisation of endometrial cancers is already being encouraged to be considered when making decisions about adjuvant treatment and provides important prognostic data (Table 1) [62]. The low rate of recurrence seen with POLE*mut* tumours suggests that an adjuvant treatment may not be required for patients with early-stage disease and a pathogenic POLE mutation [75]. On the other hand, the escalation of adjuvant treatment to include combined chemotherapy and radiotherapy appears to be beneficial for women with stage III disease and p53*abn* tumours, and this is associated with 11.3% and 22.4% increases in the 5-year failure-free survival rate, respectively, in the PORTEC-3 trial [76,77]. The absence of data to support a benefit of adjuvant chemotherapy for women with MMRd tumours, even in the presence of advanced disease, is increasingly being used to justify the avoidance of the systematic toxicity associated with its use [77].

## 6. Immunotherapy

After years of limited treatment options for advanced and recurrent endometrial cancer, the widespread approval of immunotherapy by international drug agencies (the FDA, EMA and NICE) has been heralded as a major advance for patients. Both pembrolizumab and dostarlimab bind to the PD-1 receptor and block the activation of the PDL-1 and PDL-2 pathways, which are critical in controlling immune tolerance within the tumour microenvironment. Microsatellite high (MSI-H) and MMRd tumours demonstrate the upregulation of PD-L1 and, therefore, increased sensitivity to immune checkpoint inhibition [78]. This was confirmed within the Keynote-158 study, in which a 48% objective response rate to pembrolizumab was observed in 79 patients with MSI-H/MMRd endometrial cancers, a significant proportion of whom was heavily pre-treated [79]. A similar response rate was observed with dostarlimab in this patient group (42.3%) in the GARNET trial, with an apparently sustained response [80]. Whilst both drugs are, therefore, licensed for use by women with advanced or recurrent MSI-H/MMRd endometrial cancer (Table 2) who have progressed on or following platinum-based chemotherapy, the cost may well direct drug choice, and tolerability is a concern [81,82]. Only dostarlimab has been approved for use alongside chemotherapy in the primary treatment of advanced MSI-H/MMRd endometrial cancer, following the RUBY trial, which demonstrated an improvement in progression-free survival at 24 months from 15.7% (95%CI 7.2–27.0) to 61.4% (95%CI 46.3–73.4%) with the addition of the drug [83]. The combination of pembrolizumab with Lenvatinib (a tyrosine kinase inhibitor) appears to be equally efficacious in women with MMR-proficient endometrial cancers as it does with those with MMRd tumours, resulting in a median 3.4 month prolongation of progression-free survival compared with chemotherapy alone [82]. Identifying the predictive biomarkers for this regime is now under investigation [84].

## 7. Medical Management

Increasing numbers of women are opting to undergo the medical management of their endometrial cancer, either as a means of preserving fertility or because of significant peri-operative risks driven by obesity and associated co-morbidities. Intrauterine progestin has a similar response rate to oral progesterones, but may be associated with fewer side effects, and hence, improved compliance [85,86]. Women with atypical hyperplasia are more likely to achieve a complete response than those with endometrial cancer, and molecular classification may well be used in the future to guide therapy [87,88]. Simultaneous weight loss either through dieting or bariatric surgery appears to improve the effectiveness of progesterone therapy, with women who lost > 10% bodyweight being four times more likely to respond than those who lost ≤ 10% bodyweight [89]. This may also have a beneficial effect on improving fertility, as well as reducing the risks associated with hysterectomy, particularly if there is disease relapse.

## 8. The Future of Endometrial Cancer Research

Significant advances in our understanding of endometrial carcinogenesis and the changing demographics of those diagnosed with the disease have driven seismic shifts in the way endometrial cancer is diagnosed and treated and has identified, for the first time, the ways in which this disease could be prevented. The increasing personalisation of care based on patients’ and tumour characteristics is at the heart of these advances and means that women are starting to be offered bespoke treatments designed to maximise the oncological benefits, whilst minimising the risk of harmful side effects. This is a trend that is set to continue as our understanding of the interplay between genetic and environmental influences on endometrial carcinogenesis increases.

The dramatic rise in endometrial cancer incidence over the last 30 years has emphasised the need for effective strategies for primary disease prevention, which must be targeted at those with the greatest disease risk. Adequately powered clinical trials are urgently needed to identify the most clinically and cost-effective approach to reducing disease incidence and would be supported by many national gynae-oncology groups. Improving the diagnostic pathway for individuals with postmenopausal bleeding to reduce the number requiring invasive testing is a key priority for women and is likely to benefit from ongoing advances in molecular biotechnology. The hunt is still ongoing to identify an ideal biomarker that will enable the accurate diagnosis of those with early-stage endometrial cancer, whilst offering appropriate reassurance to those without the disease. Endometrial cancer treatment in the future is likely to be determined not by traditional histological variables, but rather by the molecular profile of the tumour, with the potential for marked differences in the use of adjuvant treatments across molecular groups. Targeted therapies, including mTOR and PARP inhibitors and antiangiogenic drugs, are currently under investigation for the treatment of advanced and recurrent disease and may well have a role to play in improving survival in the future. Predictive biomarkers used to identify those who will benefit most from these often toxic and expensive therapies will be critical. Attracting funding for endometrial cancer research is essential if we are to continue to make advances in the diagnosis and treatment of the disease at pace.

## Figures and Tables

**Table 1:** Prognostic and therapeutic implications of the molecular classification of endometrial cancers.

Molecular Group	Identifying Features	Surrogate Marker	Predominant Endometrial Cancer Histotype	Prognosis (Progression Free Survival at 5 Years)	Therapeutic Implication
POLE*mut*	Very high mutational load	POLE exonuclease domain mutation	All histotypes with the exception of serous carcinomas	Excellent (92–100%)	Adjuvant treatment may not be required given excellent prognosis
MMRd	High mutational load	Loss of *MLH1*, *MSH2*, *MSH6* and/or *PMS2* expression	Predominately endometrioid carcinomas	Intermediate (80–90%)	Limited benefit from chemotherapy. Improved response to immunotherapy.
p53*abn*	Low mutational load, high copy number variations	Abnormal p53 expression	Serous, high grade endometrioid	Poor (50%)	Benefit from concurrent chemo-radiotherapy. PARP inhibitor therapy may also be effective
NSMP	Low mutational load, low copy number variations	Absence of the other markers	Low grade endometrioid	Heterogeneous but overall considered intermediate (75–80%)	May benefit from hormonal treatment given frequent ER+ and PR+

**Table 2:** Indications for immunotherapy and predictive biomarkers of response. The underlined terms signal the different licensed indications for single agent or combination therapy.

Immunotherapy Regime	Licensed Indication	Current Predictive Biomarker	Predictive Biomarkers under Investigation
Pembrolizumab	Advanced or recurrent MSI-H or MMRd endometrial cancer with disease progression following previous systemic treatment and not suitable for curative surgery or radiotherapy	MSI-H and MMRd	Tumoural immune profile (CD4, CD8 cell populations)
Dostarlimab	Single agent-advanced or recurrent MSI-H or MMRd endometrial cancer with disease progression following previous systemic treatment and not suitable for curative surgery or radiotherapy In combination with carboplatin and paclitaxel for primary advanced or recurrent MSI-H or MMRd endometrial cancer	MSI-H and MMRd	Tumour mutational burden, PD-L1 expression, ctDNA
Pembrolizumab plus Lenvatinib	Advanced or recurrent endometrial cancer, regardless of MMR and MSI status, with disease progression on or following systemic treatment and not suitable for curative surgery or radiotherapy	None	Tumour mutational burden, T cell-inflammation gene signature
